# Limited Antigenic Diversity in Contemporary H7 Avian-Origin Influenza A Viruses from North America

**DOI:** 10.1038/srep20688

**Published:** 2016-02-09

**Authors:** Yifei Xu, Elizabeth Bailey, Erica Spackman, Tao Li, Hui Wang, Li-Ping Long, John A. Baroch, Fred L. Cunningham, Xiaoxu Lin, Richard G. Jarman, Thomas J. DeLiberto, Xiu-Feng Wan

**Affiliations:** 1 Department of Basic Sciences, College of Veterinary Medicine, Mississippi State University, Mississippi State, Mississippi, the United States; 2 Exotic and Emerging Avian Viral Diseases Unit, Southeast Poultry Research Laboratory, US National Poultry Research Center, US Department of Agriculture, Agricultural Research Service, Athens, Georgia, the United States; 3 Viral Diseases Branch, Walter Reed Army Institute of Research, Silver Spring, Maryland, the United States; 4 National Wildlife Research Center, Wildlife Services, Animal and Plant Health Inspection Service, US Department of Agriculture, Fort Collins, Colorado, the United States; 5 Mississippi Field Station, National Wildlife Research Center, Wildlife Services, Animal and Plant Health Inspection Service, US Department of Agriculture, Mississippi State, Mississippi, the United States

## Abstract

Subtype H7 avian–origin influenza A viruses (AIVs) have caused at least 500 confirmed human infections since 2003 and culling of >75 million birds in recent years. Here we antigenically and genetically characterized 93 AIV isolates from North America (85 from migratory waterfowl [1976–2010], 7 from domestic poultry [1971–2012], and 1 from a seal [1980]). The hemagglutinin gene of these H7 viruses are separated from those from Eurasia. Gradual accumulation of nucleotide and amino acid substitutions was observed in the hemagglutinin of H7 AIVs from waterfowl and domestic poultry. Genotype characterization suggested that H7 AIVs in wild birds form diverse and transient internal gene constellations. Serologic analyses showed that the 93 isolates cross-reacted with each other to different extents. Antigenic cartography showed that the average antigenic distance among them was 1.14 units (standard deviation [SD], 0.57 unit) and that antigenic diversity among the H7 isolates we tested was limited. Our results suggest that the continuous genetic evolution has not led to significant antigenic diversity for H7 AIVs from North America. These findings add to our understanding of the natural history of IAVs and will inform public health decision-making regarding the threat these viruses pose to humans and poultry.

Influenza A virus (IAV) is an enveloped virus containing 8 single-stranded, negative-sense RNA genomic segments (segments 1–8) with varying lengths of 890–2,341 nucleotides. These gene segments encode at least 11 proteins: PB2, PB1 and PB1-F2, PA, hemagglutinin (HA), nucleoprotein (NP), neuraminidase (NA), matrix proteins M1 and M2, and nonstructural (NS) proteins NS1 and NS2 are encoded by gene segments 1–8, respectively. IAV serotype is determined on the basis of 2 surface glycoproteins, HA and NA. To date, 18 HA and 11 NA subtypes have been identified^1^.

IAVs evolve by 2 major mechanisms: mutation and reassortment. Point mutations within surface glycoproteins HA and NA can lead to a small antigenic change, so called antigenic drift. Reassortment refers to the exchange of individual gene segments or combinations of segments between IAVs during mixed infections in the same cell. The switch of HA and/or NA by reassortment can cause a large antigenic change, so called antigenic shift. Reassortment occurs frequently between IAVs^2^,^3^,^4^,^5^,^6^, and it facilitates generation of epidemic and pandemic influenza strains^7^,^8^. Both antigenic drift and antigenic shift allow IAVs to evade the herd immunity established from previous influenza infections or vaccination.

IAVs have a complex ecology, and waterfowl, especially migratory waterfowl, are considered the natural reservoir of IAVs. Waterfowl maintain a large genetic pool of IAVs, which contributes to emergence of novel strains that cause infections in humans, lower mammals, and domestic poultry. Through migration, waterfowl can carry IAVs from one area to another and present risks to the host species along the migratory flyway. Frequent introductions of waterfowl-origin IAVs to domestic poultry have been well documented. In Minnesota alone during 1978–2000, there were at least 108 laboratory-confirmed introductions of low pathogenic avian influenza viruses from migratory waterfowl to domestic poultry^9^. More recently, migratory waterfowl introduced highly pathogenic avian influenza (HPAI) A subtype H5 viruses from Eurasia to North America, resulting in more than 200 cases among domestic poultry in the northwestern and mid-western United States and the culling of more than 40 million birds^10^,^11^.

In addition to subtype H5, HPAI subtype H7 virus has been one of the most frequently reported subtypes and has caused sporadic outbreaks in domestic poultry and humans. The first outbreak of HPAI H7 virus in Pakistan was reported in 1995, and the threat to domestic poultry has persisted in the region since then^12^,^13^. In 2003, a subtype H7N7 HPAI virus outbreak in the Netherlands led to the death or culling of more than 30,000,000 birds and 89 infections in humans, 1 of which was fatal^14^,^15^. In March 2013, a low pathogenic avian influenza subtype H7N9 virus emerged in eastern China^16^ and has become enzootic in the region^17^. The virus causes asymptomatic infection in domestic poultry, including chickens and waterfowl, but high morbidity and mortality in human infections^18^. Since the emergence of H7N9 virus, more than 500 laboratory-confirmed cases in human have been reported, of which more than 100 were fatal (http://www.who.int/influenza/human_animal_interface/influenza_h7n9/en/). Furthermore, epidemics caused by H7N1 and H7N3 avian influenza viruses (AIVs) were reported in Italy during 1999–2004^19^,^20^. H7 AIVs were also reported to cause outbreaks in domestic poultry in Australia^21^, Germany^22^, and the United Kingdom^23^. In North America, H7N2 AIV was first identified in 1994 in the live poultry market system in the northeastern United States, and during 1997–2002, it was linked with outbreaks among poultry in Pennsylvania, Virginia, West Virginia, and North Carolina^24^,^25^. HPAI H7N3 viruses were responsible for outbreaks in poultry in Canada (in 2004 and 2007), and Mexico (in 2012)^26^,^27^,^28^ and subsequently spilled over to humans^29^,^30^.

The conventional strategy for controlling the spread of avian influenza outbreaks in domestic poultry involves enforcement of biosecurity measures, diagnostics and surveillance, and culling of infected birds. In addition, vaccination programs have been implemented in multiple countries to control H7 AIV outbreaks among domestic poultry. For example, in Italy, vaccine was used against H7N1 virus in 2000 and against H7N3 virus in 2002; vaccines have been used in Pakistan since 1995 to control H7N3 virus; and in North America, vaccine was used against a 2003 H7N2 virus outbreak in Connecticut, United States, and against the on-going H7N3 virus outbreak in Mexico. The use of influenza vaccine can reduce or prevent clinical disease; reduce or eliminate virus shedding into the environment from infected birds, which would help prevent the spread of virus to uninfected flocks; and increase the resistance of birds to becoming infected. Early experience showed that vaccine could serve as a valuable component in a successful AIV control strategy.

Understanding the antigenic diversity of H7 AIVs circulating in North America will facilitate not only our understanding of the natural history of IAVs but also the detection of influenza virus antigenic variants and development of effective strategies for disease prevention and control. In this study, we antigenically characterized 93 subtype H7 influenza isolates derived from migratory waterfowl, domestic poultry, and a seal; the isolates were collected across North America during 1971–2012. The genomic sequences were analyzed to determine the genetic evolution dynamics of H7 AIVs in North America.

## Materials and Methods

### Ethics statement

All experiments involved in animals were approved by Institutional Animal Care & Use Committee, Mississippi State University (Project No. 13–090). All experiments were carried out in accordance with the approved guidelines.

### Viruses

**Figure 1:**
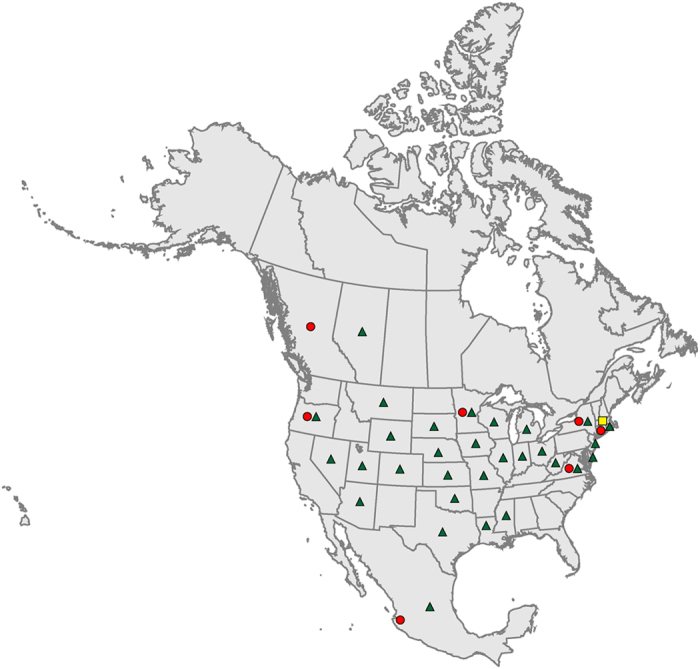
Geographic distribution of subtype H7 AIVs selected for antigenic characterization. The isolates represent strains from Canada, Mexico, and 30 states in the United States. Tringles indicate viruses isolated from migratory waterfowl, dots indicate viruses isolated from domestic poultry, and the square indicates virus isolated from a seal. Map of North America with US States and Canadian Provinces by FreeVectorMaps.com, https://freevectormaps.com/world-maps/north-america/WRLD-NA-02-0003.

A total of 93 H7 isolates were included in the study (Table 1): 85 were derived from migratory waterfowl during 1976–2010, 7 were derived from domestic poultry during 1971–2012, and 1 was derived from a seal in 1980. Among these isolates, A/cinnamon teal/Mexico/2817/2006(H7N3) was the vaccine strain used during the vaccination campaign against HPAI subtype H7N3 virus in Mexico in 2012, strain A/chicken/British Columbia/314514-2/2004(H7N3) was isolated from the outbreak in domestic poultry in Canada in 2004, and strain A/chicken/Jalisco/CPA-12283/2012(H7N3) was from the outbreak in Mexico in 2012. The waterfowl-origin isolates represent those recovered from Canada, Mexico, and 28 US states. They also represent the 4 major bird migratory flyways in North America: the Atlantic flyway, Central flyway, Mississippi flyway, and Pacific flyway (Fig. 1).

The isolates were propagated by using 9- day-old specific pathogen–free (SPF) chicken embryonated eggs; the eggs were inoculated and incubated for 72 hours at 37 °C before the virus was harvested. Viruses were then aliquoted and stored at −80 °C until use.

### Generation of reference antisera in chicken

A total of 15 H7 isolates were selected to generate reference antisera; the isolates were selected to maximize the representative subtype and species diversity and geographic and temporal coverage of the 93 isolates (Table 1). Three-week-old SPF chickens were used to produce antisera. Chickens were inoculated intranasally with 10^6^ 50% tissue culture infective doses of an H7 AIV isolate. If the sera titers were ≥ 1:160 at two weeks post inoculation, the sera were collected at three weeks post inoculation; if the viral titers were < 1:160 at two weeks post inoculation, the birds were re-inoculated intranasally with 10^6^ 50% tissue culture infective doses of the same H7 AIV isolate and the sera were collected at two weeks post re-inoculation. Blood was collected from the chickens’ heart 4 weeks after the first inoculation. Serum was separated from the erythrocytes after centrifugation at 2,000 rpm for 10 minutes. All sera were aliquoted and stored at −80 °C until use.

### HA and hemagglutination inhibition (HI) assays

Before performing the HI tests, we treated the chicken antisera with 100% packed chicken red blood cells to eliminate non-specific antigen reactions. The HA and HI assays were performed in accordance with World Organisation for Animal Health guidelines^31^. HI tests were carried out by using 4 hemagglutinin units and a 1% chicken red blood cells suspension.

### Full genome sequencing

Viral RNA was extracted from the allantoic fluid of SPF embryonated chicken eggs by using the QIAamp Viral RNA Kit (QIAGEN, Valencia, CA) according to the manufacturer’s instructions. The full-length cDNA for 8 influenza gene segments was amplified by using a SuperScript One-Step RT-PCR kit (Invitrogen, Grand Island, NY) with influenza virus–specific primers^32^. PCR products were separated by using agarose gel electrophoresis and purified by using a QIAquick Gel Purification Kit (QIAGEN). Amplified viral DNA products were quantitated by using a High Sensitivity DNA kit on an Agilent 2100 Bioanalyzer system (both from Agilent Technologies, Santa Clara, CA). An equal amount of each ample was used to prepare the sequencing library with the Illumina Nextera DNA Sample Preparation Kit (Illumina, San Diego, CA, USA). Library samples were further quantitated, normalized, and pooled together. Pooled library samples were sequenced by using a MiSeq Reagent Kit v2 (500 cycles) on a MiSeq sequencer (Illumina); the sequencing protocol suggested by the manufacturer was followed. When any gene segment presented more than 1 copy of sequence, PCR was performed with specifically designed primer to confirm the existence of multiple copies of sequences. These sequences were excluded from further analysis. Sequences obtained in this study are available in GenBank under accession numbers KU289738 to KU290331.

### Genomic assembly

Genomic assembly was conducted with the in-house influenza genome assembly pipeline, which integrates quality trimming by Trimmomatic^33^, *de novo* assembly by Velvet^34^, reference search by BLAST^35^, and mapping by Bowtie v2.0^36^. In brief, quality trimming was first conducted by using Trimmomatic, which trims bases from both ends of each read if the quality falls below 30 and clips reads if the average quality drops below 28 (in a sliding window of 10 bases). Reads less than 100 nt in length were not included in the downstream analyses. The quality-filtered reads were then *de novo* assembled in Velvet to build long contigs, and assembled contigs were searched against the Influenza Virus Resource^37^ by using BLAST to select the reference sequence. Quality-filtered reads were then mapped to the reference sequences by using Bowtie 2. Last, consensus sequences were generated with a minimum 10-fold mapping coverage and supported by at least 90% of reads at a given position. The mapping profile was visualized by using the Integrative Genomics Viewer^38^ and manually checked to correct potential assembly errors.

### Evolutionary analysis

Phylogenetic trees were inferred by using the maximum-likelihood method implemented in RAxML v8.1.17^39^. A general time-reversible model of nucleotide substitution and a γ-distributed rate variation among sites was applied throughout the analysis. Sequence alignments were conducted by using MUSCLE v3.8^40^.

To understand the evolutionary history of the H7 gene of AIVs from North America, 2 rounds of phylogenetic analyses were conducted. In the first round, all genomic sequences of the H7 gene from AIVs were downloaded from the Influenza Virus Resource in March 2015. A total of 1,315 sequences were available after combining these database sequences with sequences recovered in this study. Two major lineages, North American and Eurasian, were identified from the topology of this preliminary phylogeny. Sequences falling into the North American lineage were kept, and 19 sequences were selected to represent the Eurasian lineage. After that, 775 sequences were retained. In the second round, phylogenetic analysis was conducted on the HA1 domain of these 775 nucleotide sequences for a clear comparison of the antigenic and genetic profiles. Topological robustness of the tree was evaluated by 1,000 pseudo-replicates.

Two rounds of phylogenetic analysis were conducted to assess the evolutionary history of the 6 internal gene segments of H7 AIVs circulating among wild birds in North America. In the first round, 8,545 genomic sequences from 1,030 complete genomes of H7 AIVs were downloaded from the Influenza Virus Resource. When multiple copies of genomic sequences were present for 1 gene segment, the sequence corresponding to the maximal length was reserved. These database sequences were analyzed with complete genomes recovered in this study. A total of 1,098 complete genomes were retained, and phylogeny was inferred for each gene segment. Topology of the phylogeny was supported by 100 pseudo-replicates. North American and Eurasian lineages were identified from the tree topology. In the second round of analysis, the phylogenetic tree was inferred for 316 complete genomes corresponding to wild bird–origin isolates in the North American NA-WB lineage. Topology of the phylogeny was supported by 1,000 pseudo-replicates. Genotypes were defined on the basis of the phylogeny of each gene segment. A monophyletic clade was identified by two criteria: (1) it was supported by a bootstrap value above 60, and (2) all sequences in the clade had an average genetic distance greater than 95%.

The extent of reassortment among the internal gene segments was determined on the basis of the 6 gene segment phylogeny for viruses in the NA-WB lineage. A maximum likelihood method was implemented to measure the congruence among these trees. Each gene segment tree was fitted to 6 gene data sets in turn, and the log likelihood value was obtained after optimizing model parameters and branch lengths. The similarity in topology among 6 gene trees corresponding to the same dataset was determined by the difference in log likelihood values. To put the distribution of log likelihood value in context, 100 random trees were generated for each gene dataset. Log likelihood values were obtained, using the same approach, after fitting them to the reference gene dataset.

### Inference of amino acid sides under positive selection

Selective pressure for the HA gene segment was investigated by using codon substitution models implemented in the Codeml program of PAML v 4.8^41^. The site models were used, allowing the ratio of nonsynonymous/synonymous substitution rates (d_N_/d_S_) to vary among sites. Four different models were used: M1a, M2a, M7, and M8. Likelihood ratio tests for two pairs of models (M2a versus M1a and M8 versus M7) were conducted according to instructions in the manual. In the test, twice the log likelihood difference between the alternative model and null model was calculated. Small p-values ( < 0.01) lead to the rejection of the null models. If null models were rejected, then Bayes Empirical Bayes analysis was implemented for estimation of specific codons under positive selection. Before analysis, identical sequences and sequences with ambiguous positions were removed. The nucleotide sequences coding the HA1 protein were analyzed.

### Molecular dating

Molecular dating was conducted by using the Bayesian Markov Chain Monte Carlo method implemented in BEAST v1.8.0^42^. A HKY85 nucleotide substitution model, Bayesian skyline coalescent tree prior, and relaxed uncorrelated lognormal clock model were applied. For each analysis, a chain length of 50 million steps was run and 2,000 samples were generated. The results were analyzed in Tracer v1.6 (http://tree.bio.ed.ac.uk/software/tracer/), and convergence was assessed with a cutoff of 200 for the effective sample size. The mean nucleotide substitution rate and time to the most common ancestor were computed after 10% of the samples were removed as burn-in, and the statistical uncertainty was evaluated by using the values of the 95% highest posterior density (HPD).

### Construction of the antigenic cartography and molecular characterization

Antigenic cartography was constructed on the basis of HI data by using AntigenMap (http://sysbio.cvm.msstate.edu/AntigenMap)^43^. Each entry in the HI table was normalized by dividing the maximum HI value for the reference antiserum. Missing HI titers and those below the cutoff value for low reactors were analyzed by low-rank matrix completion. An HI titer of 10 was used as the low-reactor cutoff in the HI assay. Antigenic distance between 2 antigens was defined as the Euclidean distance between the HI values of the 2 viruses against all the antisera. Each unit of the antigenic distance corresponded to a 2-fold change in HI titer. Multidimensional scaling was used to project viruses to a 2-dimensional map by minimizing the sum-squared error between map distance and antigenic distance. Antigenic distance was subjected to hierarchical clustering analysis implemented in R (https://www.r-project.org/) to determine the potential division of viruses.

To investigate the difference in antibody binding site within the HA sequences, the protein sequences of these isolates were aligned with the H3 protein sequences and the antibody binding sites were annotated based on those in H3N2 influenza A virus as shown previously^44^. 135 amino acid positions corresponding to five antibody binding sites A, B, C, D, and E in the H3 protein were identified.

## Results

### H7 HA genes are genetically diverse

Phylogenetic analyses showed that HA gene of H7 AIVs was divided into 2 geographically dependent lineages: Eurasian and North American (Fig. S1). Sporadic intercontinental gene flow between these 2 genetic pools was observed. Five H7 viruses isolated in North America fell into the Eurasian lineage, and at least 2 independent introductions (in 1992 and 1994) were identified. AIVs A/softbill/CA/33445-158/1992(H7N1) and A/softbill/California/33445-136/1992(H7N1) were most closely related to A/non-psittacine/England-Q/1985/89(H7N7), sharing nucleotide sequence identities of 96.3% and 96.4%, respectively. In addition, A/softbill/California/13907-21/1994(H7N1) and A/Pekin robin/California/30412/1994(H7N1) are closely related to a group of AIVs of the same subtype isolated from migratory waterfowl in the Netherlands, Singapore, and England in 1994; nucleotide sequence identities ranged from 97.8% to 99.4%. One H7 virus isolated in China, A/duck/Guangdong/1/1996(H7N3), was grouped with waterfowl-origin viruses from North America and was genetically closely related to A/ruddy turnstone/Delaware Bay/135/1996(H7N3), sharing a nucleotide sequence identity of 99.4%.

Three major genetic clusters were identified in the North American lineage. Cluster I comprised the viruses isolated from waterfowl and domestic birds during the 1970s into the early 1990s. From the available data, a clear temporal division was observed around 1994: cluster II mainly comprised the domestic poultry-origin H7 viruses isolated during1994–2006, and cluster III comprised the viruses recovered from waterfowl and domestic poultry after 1993. The tree topology was supported by the sequence identities: the average shared identity between HA1 nucleotide sequences within these clusters was 94.0% (cluster I), 96.1% (cluster II), and 95.6% (cluster III), and the average identities between viruses from 1 cluster and another were 88.0% (clusters I and II), 89.5% (clusters II and III), and 89.4% (clusters I and III) (Table 2).

Cluster I was estimated to emerge around 1969 (Table 3), and it circulated in North America for 24 years before its extinction in 1993. This cluster included 1 isolate recovered from a seal in 1980. Subtype H7N2 was the predominant subtype in cluster II, and the H7N2 viruses were mainly isolated from the live-poultry market system in the northeastern United States and from commercial poultry farms in 4 US states (Maryland, Pennsylvania, North Carolina, and Virginia). Cluster II was further divided into 2 clades: II-1 and II-2. Clade II-1 mainly consisted of viruses isolated during 1994–1996, and clade II-2 consisted of viruses isolated during 1996–2006. A clear molecular difference separated these 2 clades: a deletion of 8 amino acids at positions 212–219 in the HA1 protein. Cluster II included A/New York/107/2003(H7N2), which infected a human with suspected exposure to poultry^45^. A similar 8–amino acid deletion was also observed in the HA1 protein of this virus.

Cluster III represents the contemporary genetic pool of H7 AIVs in North America, including viruses from migratory waterfowl and from outbreaks among domestic poultry. H7 viruses isolated during individual outbreaks among domestic poultry formed independent monophyletic clades in the phylogeny, suggesting that they originated from separate introductions from waterfowl^46^. Three of the H7 viruses evolved into HPAI viruses: A/chicken/Canada/314514-2/2005(H7N3), A/chicken/SK/HR-00011/2007(H7N3), and A/chicken/Jalisco/CPA1/2012(H7N3). Another H7 introduction, A/chicken/Delaware/10851/2014(H7N7), did not evolve into an HPAI virus.

### Limited antigenic diversity among tested H7 AIVs

**Figure 2:**
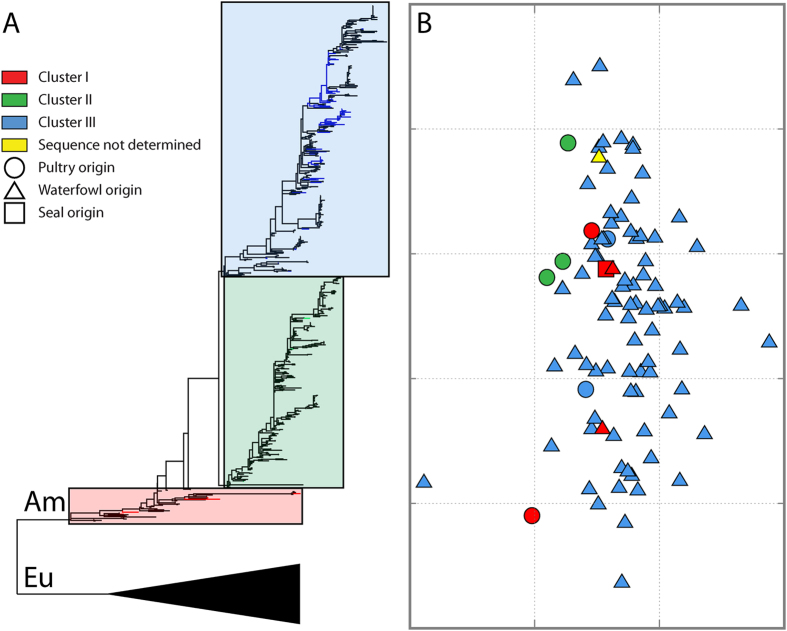
Comparison of genetic and antigenic evolution of subtype H7 AIVs from North America. (**A**) Schematic phylogenetic tree of the HA1 nucleotide sequences of H7 AIVs; the tree was constructed on the basis of the phylogeny inferred by using the maximum-likelihood method as shown in Fig. S1. Boxes represent the 3 major genetic clusters; branches corresponding to viruses subjected to antigenic characterization are colored-coded as described in the key; the same color code is used in (**B**). The Eurasian lineage (EU) is represented by the large black triangle. Am = North American lineage. (**B**) Antigenic cartography constructed (AntigenMap, http://sysbio.cvm.msstate.edu/AntigenMap) on the basis of HI data in Table S1. Each gridline (horizontal and vertical) represents a 2-fold difference in HI titer.

Serologic analyses showed that the antisera generated against 15 selected isolates cross-reacted with the tested H7 isolates to different extents (Table 4, Table S1). Antigenic cartography showed no clear division of these isolates (Fig. 2B). The average antigenic distance among these isolates was 1.14 units (SD, 0.57 unit), and each unit represented a 2-fold change in HI titer. The hierarchical clustering method grouped these viruses into 1 cluster at a distance of 1.46 units, except for 1 outlier, A/laughing gull/NJ/2455/2000(H7N3). The average distance from the outlier to the remaining isolates was 2.01 units. Viruses from 3 distinct genetic clusters were grouped in this antigenic cluster; no clear correlation between the genetic diversity and antigenic property was observed (Fig. 2A,B).

Viruses isolated from domestic poultry and waterfowl lacked antigenic diversity. The average antigenic distance among waterfowl-origin isolates was 1.14 units (SD, 0.57 unit). With the exception of 1 outlier, A/laughing gull/NJ/2455/2000(H7N3), hierarchical clustering showed that these isolates merged into 1 cluster at a distance of 1.47 units. The average antigenic distance among 7 poultry-origin isolates was 1.13 units (SD, 0.71 unit). These viruses were grouped into 1 cluster, at a distance of 1.63 units, by the hierarchical clustering method. Poultry-origin isolates were antigenically similar to those from waterfowl; the distance between poultry-origin isolates and the most antigenically similar waterfowl-origin isolates was 0.18–0.86 units.

Limited antigenic diversity was supported by the comparison of amino acid sequence of the 135 residues corresponding to those in the reported antibody binding sites in influenza HA protein. Results showed that the average shared identity among tested isolates was 96.6%, and lack of divergence of these amino acid positions (Fig. S2, Table S2).

### Genetic evolution dynamics of the HA gene

**Figure 3:**
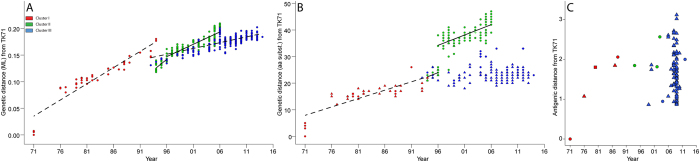
Comparison of temporal genetic and antigenic evolution dynamics for subtype H7 AIVs from North America. (**A**) Year-by-year analysis of genetic evolution dynamics. Genetic distances between each virus and A/turkey/Oregon/1971 (TK/71) were determined from the maximum-likelihood (ML) phylogenetic tree in Fig. S1. Dashed line indicates linear regression fit for viruses in genetic clusters I and III; solid lines indicate linear regression fit for 2 clades in genetic cluster II. (**B**) Same as panel A, but genetic distances are characterized by the number of amino acid substitutions (aa subst.) in the HA1 protein. (**C**) Year-by-year analysis of antigenic evolution dynamics. Antigenic distances were calculated from antigenic cartography. 71 (1971), and 16 (2016). In panel B and C, dots indicate viruses isolated from domestic poultry, triangles indicate viruses isolated from waterfowl, the square indicates virus isolated from a seal, and crosses indicate virus isolated from human.

To evaluate the genetic evolution dynamics for the HA gene of H7 AIVs from North America, we analyzed (in temporal order) the genetic distance from each virus to A/turkey/Oregon/1971(H7N3), the oldest isolate in the phylogeny (Fig. 3A). The average evolutionary rate on the nucleotide level was determined by the slope of the linear regression line that fits the data. Viruses in cluster I demonstrated a gradual and stable increase of genetic distance to A/turkey/Oregon/1971(H7N3). The regression line for cluster I had a slope of 0.0061 (adjusted R^2^ = 0.88; P < 2.20E-16). The lack of continuations from 1971 to 1976 was due to the limited sampling during that time period. Viruses in cluster II evolved at a faster rate than those in cluster III (0.0052 [adjusted R2 = 0.82; P < 2.20E-16] vs. 0.00218 [adjusted R^2^ = 0.42; P < 2.20E-16], respectively). Cluster II was separated into 2 clades at the 1996 time point, and an elevated increase in genetic distance was identified at that time. To determine the fluctuation of evolutionary dynamics in cluster II, we independently analyzed viruses in 2 clades. Regression analysis showed that viruses in clade II-1 evolved faster than those in clade II-2 (0.008 [adjusted R^2^ = 0.59; P < 4.07E-9] vs. 0.0046 [adjusted R^2^ = 0.67; P < 2. 20E-16], respectively). Cluster III consisted of viruses isolated from waterfowl and individual outbreaks among domestic poultry. To more precisely evaluate the evolutionary status for H7 AIVs in the natural reservoir, we conducted additional analyses for waterfowl-origin viruses in this cluster. The slope of the regression line was 0.0019 (adjusted R^2^ = 0.31; P < 2.20E-16), lower than that for the entire population.

Similar analyses were conducted at the amino acid level by characterizing the genetic distance by the number of amino acid substitutions (Fig. 3B). A consistent evolution trend was observed for cluster I, which showed an average of 0.63 amino acid substitutions per year (adjusted R^2^ = 0.70; P < 3.27E-14). Cluster II had an average change of 1.47 amino acids per year (adjusted R^2^ = 0.71; P < 2.20E-16). A dramatic increase in the number of amino acid substitutions was observed in 1996; this finding is consistent with the observed deletion of 8 amino acids in the HA1 protein at the same time. However, viruses in clades II-1 and II-2 had a similar average rate of evolution. The slopes of regression lines for these 2 clades were 0.80 (adjusted R^2^ = 0.26; P < 0.00044) and 0.78 (adjusted R^2^ = 0.46; P < 2.20E-16), respectively. The temporal fluctuation of amino acid substitutions for viruses in cluster III was not well fitted by the linear regression model. However, it was observed that the number of amino acid substitutions in viruses isolated from poultry and humans is larger than that in waterfowl-origin viruses.

Rates of mean nucleotide substitution and the time to the most recent common ancestor were also estimated by using the Bayesian Markov Chain Monte Carlo method (Table 3). The mean evolutionary rate for the cluster I was estimated as 5.11 × 10^−3^ substitutions per site per year (sub/site/year) (95% HPD, 3.94–6.25 × 10^−3^). Cluster II evolved at a higher mean evolutionary rate (5.34 × 10^−3^ sub/site/year; 95% HPD, 4.68–6.11 × 10^−3^) than cluster III (4.84 × 10^−3^ sub/site/year; 95% HPD, 4.34–5.31 × 10^−3^). For cluster II, the estimated evolutionary rate for a collection of viruses in clade II-2 decreased to 4.90 × 10^−3^ sub/site/year (95% HPD, 4.19–5.67 × 10^−3^). This result suggested that cluster II experienced a higher evolutionary rate during 1994–1996 and a lower evolutionary rate afterwards. For cluster III, the estimated evolutionary rate for a collection of waterfowl-origin viruses was 4.60 × 10^−3^ sub/site/year (95% HPD, 4.10–5.11 × 10^−3^), which is lower than that for the entire cluster III population.

The corresponding analysis of antigenic evolution dynamics showed that antigenicity was relatively stable during 1971–2012 (Fig. 3C). No clear correspondence was observed between the genetic and antigenic evolution dynamics. Of the 93 characterized viruses, 72 were isolated in 2008 and 2009; the antigenic distances between these viruses and A/turkey/Oregon/1971(H7N3) ranged from 0.86 to 3.12 units. The antigenic distances between the other 21 isolates and A/turkey/Oregon/1971(H7N3) also fall into this range.

### Natural selection for HA gene of H7 AIVs in North America

The selective pressure for the HA gene was investigated independently for each of the 3 genetic clusters (Table 5). The mean d_N_/d_S_ for cluster II (d_N_/d_S_ = 0.1875–0.2154) was higher than that for cluster III (d_N_/d_S_ = 0.1232–0.1407). The HA gene of viruses in clade II-2 had a lower mean d_N_/d_S_ (0.1821–0.2121) than the entire population in cluster II. This finding suggests that the purifying selection pressure for cluster II was lower during 1994–1996 than afterwards. Waterfowl-origin isolates in cluster III were considered separately, and the results showed that the mean d_N_/d_S_ was lower than that for the entire population in cluster III. This finding suggests that the purifying selection pressure was greater for waterfowl-origin viruses than for poultry-origin viruses. Overall, the HA gene of H7 AIVs circulating in North America is under strong purifying selection, although there is variation in selective pressure for distinct genetic clusters.

Amino acid position 189 (H3 numbering; H7 numbering, 180) in the HA1 protein was found to be under positive selection for the waterfowl-origin viruses in cluster III. The amino acid profile of this position for H7 AIVs from Eurasia and North America was analyzed. Viruses isolated from land-based poultry and waterfowl were considered separately (Table 6). For viruses in the North American lineage, position 189 was found to be highly polymorphic in genetic clusters I and III: 3 and 5 distinct amino acids were observed, respectively, and T was the major amino acid in both clusters. HA gene in cluster II showed a distinct and conserved profile for position 189; with only one exception, S was the predominant amino acid. Amino acid profiles were also analyzed for 3 H7 viruses isolated from humans in North America: A/NewYork/107/2003(H7N2), which is from cluster II, and A/Mexico/InDRE7218/2012(H7N3) and A/Canada/rv504/2004(H7N3), which are from cluster III. Amino acid S was observed at position 189 in A/NewYork/107/2003 (H7N2), and amino acid T was observed in the other 2 viruses. Amino acid S was not observed at position 189 of Eurasian lineage H7 AIVs.

### Frequent reassorment of internal genes of H7 AIVs in NA-WB lineage

**Figure 4:**
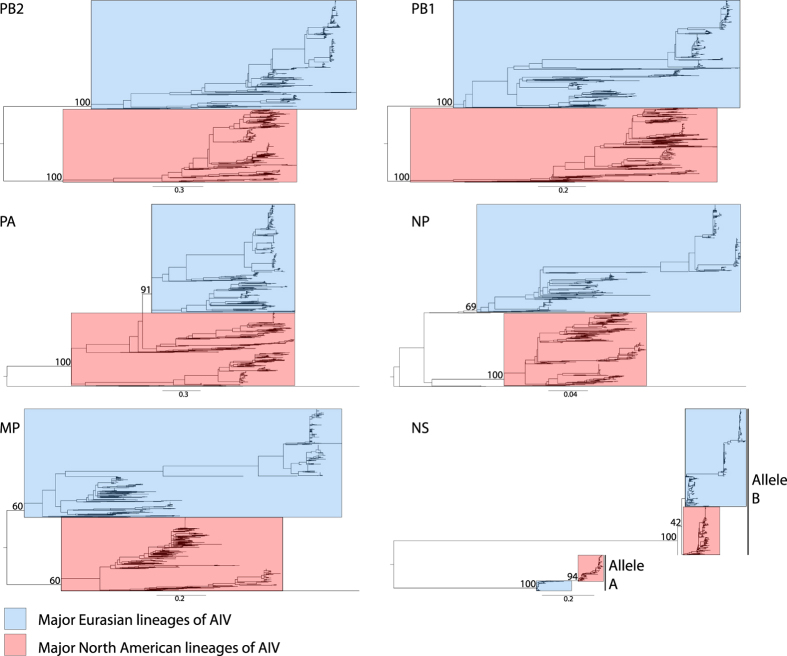
Maximum-likelihood phylogenies for internal gene segments of subtype H7 AIVs of Eurasian and North American lineage. Black bars on the lower right indicate 2 alleles in the NS gene phylogeny. Bootstrap values estimated from 100 resamplings of the sequence data are shown adjacent to selected nodes.

**Figure 5:**
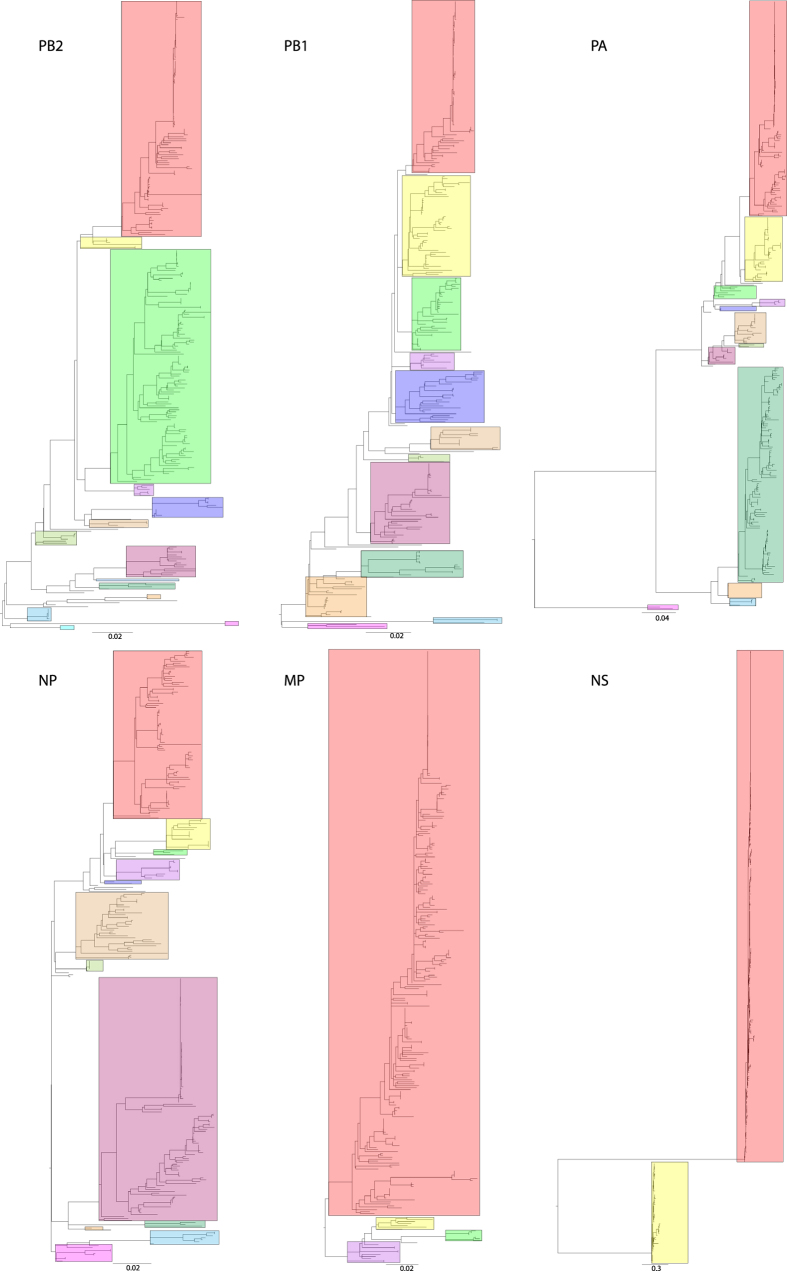
Maximum-likelihood phylogenies for internal gene segments of subtype H7 AIVs derived from wild birds from North America. Boxes represent the genetic clades. Scale bars indicate substitutions per site.

Panoramic phylogenetic analyses of all H7 complete genomes showed a clear division of the 2 major lineages (North American and Eurasian) for each gene segment (Fig. 4). It is noteworthy that the topology of the NS gene segment phylogeny showed a deep divergence between the A and B alleles: within each allele, virus isolated from North America and Eurasia was separated. Phylogenetic analysis of internal genes in the NA-WB lineage demonstrated a high level of heterogeneity (Fig. 5). Multiple distinct clades could be identified for each gene segment. The largest number of clades was observed in the PB2 phylogeny, which had 14 clades. Phylogeny of PB1, PA, and NP genes could be separated into 12 clades. Less genetic diversity was observed for the matrix protein and NS genes, which had 3 and 2 distinct clades, respectively.

**Figure 6:**
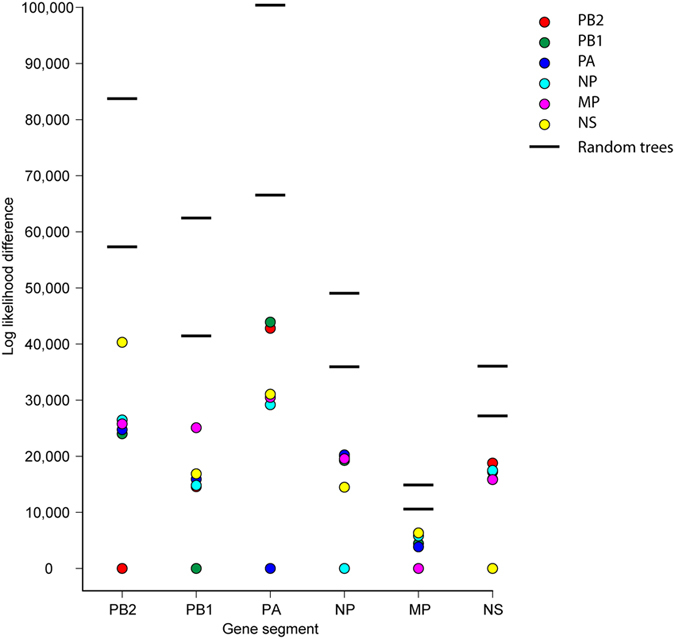
Congruence in topology among 6 internal gene segment phylogenies for subtype H7 AIVs derived from wild birds in North America. Each column represents the difference in log likelihood value when 6 internal gene phylogenies and 100 random phylogenies were fitted to the same dataset. The difference in log likelihood value for each gene phylogeny is indicated by a colored dot. Range for random phylogenies is represented by 2 solid lines.

The frequencies and patterns of reassortment of the 6 internal genes were assessed by determining the congruence among each gene’s phylogenetic tree. Results showed that the topologies of the internal gene trees were more similar to each other than to 2 random phylogenetic trees (Fig. 6), suggesting that the internal gene segments are not completely independent from each other. However, the dissimilarities in tree topology were extensive. The most significant incongruence was observed between NS gene and other gene segments; the NS gene tree was found to be closer in topology to random trees rather than to the other 5 internal gene trees. These results indicate frequent reassortment of the internal genes for H7 AIVs in lineage NA-WB; no clear link among specific gene segments was observed.

**Figure 7:**
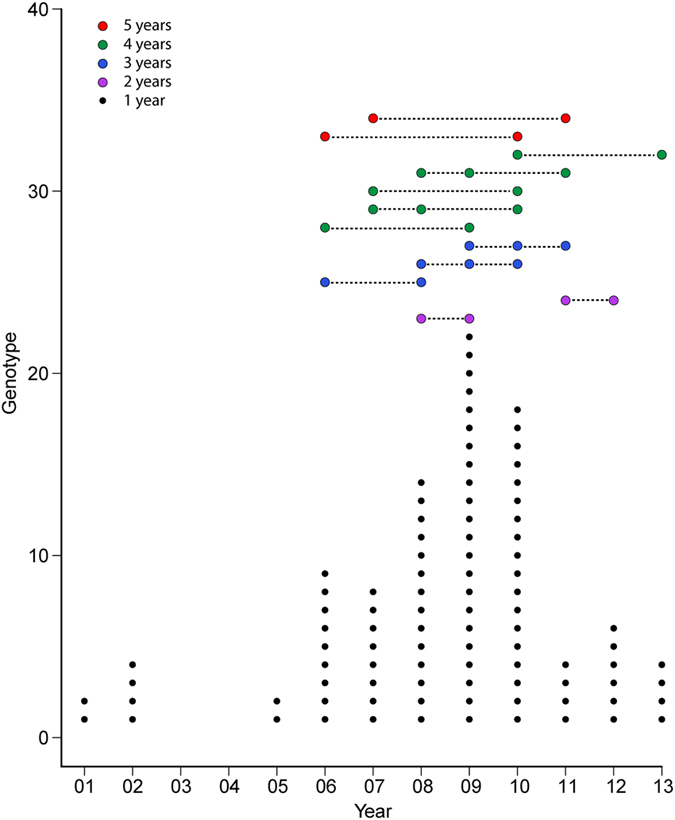
Year-by-year analysis of the evolution dynamics of internal gene constellations for subtype H7 AIVs derived from wild birds in North America during 2001–2013. Small black dots indicate constellations that existed only 1 year. Larger colored dots indicate constellations that were observed in multiple years; the span of years is indicated by a dashed line between the dots, and colors indicate the number of years constellations existed.

We assigned genotypes to viruses in the NA-WB lineage and analyzed the dynamics of these genotypes. Because 91% of the viruses were isolated during 2001–2013, our analysis was constrained to viruses from this time period. H7 AIVs in the NA-WB lineage demonstrated diverse genotypes: we identified 104 distinct genotypes for the internal gene constellation (Fig. 7). Multiple genotypes were observed in the same year, and the largest number of genotype (29) was observed in 2009. Internal gene constellations were transient rather than stable, and no individual genotype existed throughout 2001–2013. A total of 92 genotypes existed for only 1 year, and 2 genotypes existed for the maximal time span of 5 years.

## Discussion

We genetically and antigenically characterized 93 H7 AIV isolates from North America. Our results show that H7 AIVs in North America have wide genetic diversity. Gradual accumulation of nucleotide and amino acid substitutions is observed for the hemagglutinin of H7 AIVs from waterfowl and domestic poultry. Our results also show a limited antigenic diversity among the H7 viruses we tested.

In waterfowl, the limited antigenic diversity for H7 AIVs is consistent with the concept of evolutionary stasis^47^. These results are consistent with those from other studies of waterfowl-origin H7 viruses in other regions. For example, a 2005 study showed that 4 H7 AIVs isolated from mallards in Sweden and the Netherlands in 2000 and 2002 had relatively conserved antigenic properties^48^. In addition, antigenic differences between these mallard-derived H7 isolates and an H7N7 HPAI strain isolated in the Netherlands in 2003 were within a 4-fold change. No significant antigenic differences (i.e., within a 4-fold change) were observed for 9 H7 AIVs isolated in Italy and China during 1999–2005^49^; of the 9 isolates,4 were H7N7 viruses derived from ducks in China in 2003, 3 were H7 isolates derived from mallards in Italy during 2001–2005, and 2 were H7 viruses derived from turkeys in Italy in 1999 and 2002.

Our molecular characterization results suggest that the genetic evolution pattern in the isolates we tested is gradual and stable. The estimated mean rate of evolution for the H7 gene of AIVs circulating among waterfowl in North America is 4.60 × 10^−3^ sub/site/year, which is similar to the rate for Eurasian lineage H7 viruses (5.75 × 10^−3^ sub/site/year)^17^. Selection analysis showed that the H7 gene is under strong purifying selection in waterfowl; however, no amino acids at known antibody binding sites were under positive selection. The limited antigenic diversity among the H7 isolates might be attributable to the lack of herd immunity pressure in migratory waterfowl due to the absence of certain pattern recognition receptors. These receptors are triggered by influenza virus and could initiate the activation of innate immune responses. A 2010 study^50^ showed that the presence of the retinoic acid–inducible gene 1 (RIG-I) in ducks induces the production of IFN-β and expression of downstream IFN-stimulated antiviral genes; however, the absence of RIG-I in chickens contributes to their increased susceptibility (compared with that of ducks) to influenza virus. In addition, Toll-like receptors (TLR) induce the expression of type I IFN and proinfammatory cytokines^51^. TLRs 3,7,8, and 9 upregulate in naturally occurring influenza, and they are associated with innate virus inhibitory and proinflammatory responses^52^. TLRs in birds differ from those in mammals. Furthermore, TLR9 is absent in avian species, and many TLRs in waterfowl have yet to be identified^53^.

Vaccination is a key component of the control strategy for HPAI viruses. However, antigenic variations in AIVs from domestic poultry have been reported, and these variations seem to be caused by the use of vaccine in poultry flocks. In 2002, poultry flocks across Italy were vaccinated to prevent an outbreak of H7N3 virus; the vaccine contained an inactivated H7N1 AIV (i.e., a vaccine with the same HA subtype as the outbreak virus but with an antigenically and genetically different NA subtype). Antigenic characterization of a longitudinal collection of 41 isolates showed large antigenic difference between viruses isolated before and after implementation of the vaccination program^44^. Antigenic change was mirrored by the simultaneous appearance of 4 amino acid substitutions within the antibody binding sites, and 1 amino acid was positively selected after the use of vaccine. In addition, the poultry vaccination program in Mexico has facilitated the antigenic evolution of HPAI H5N2 virus in that country. An outbreak of HPAI H5N2 virus was detected in 1994 in Mexico, and a vaccination program, using inactivated vaccine, was implemented in 1995. Phylogenetic analysis of 52 H5N2 viruses isolated during 1993–2002 showed that 2 genetically different sublineages had emerged after introduction of the vaccine program and replaced the early sublineages^54^. Viruses in these 2 new sublineages had undergone antigenic drift and acquired a more than 4-fold antigenic change from the vaccine strain. Six amino acid substitutions located within antibody sites A, B, and C were detected for these 2 novel sublineages. Similar changes were observed for the emergence of an H5N1 influenza variant in China. Since September 2005, routine vaccination programs against H5N1 virus in domestic poultry have been conducted nationwide in China. However, results of serologic testing of serum samples from 1,113 chickens in Guangdong and Guizhou, China, suggested that among the vaccinated poultry, protection against an FJ-like H5N1 variant was poor compared with that against other co-circulating H5N1 viruses^55^. The presence of vaccine pressure probably selected the FJ-like H5N1 viruses, and these viruses became predominant in the region.

The change of antigenicity in these outbreaks was mirrored by the observed fast rate of evolution. The H7N3 AIVs from the Italian epidemic were estimated to evolve at a mean rate of 8.04 × 10 × 10^−3^ sub/site/year, whereas the evolutionary rates for 2 emerging H5N2 sublineages in Mexico were estimated to be 12 × 10^−3^ and 10 × 10^−3^ sub/site/year, respectively. These evolutionary rates are significantly higher than those reported for H7 and H5 viruses from domestic poultry in the absence of vaccine program, as exemplified by the range of evolutionary rates for H5N1 viruses isolated from Thailand, Turkey, and Nigeria: 2.5 × 10^−3^ to 5.2 × 10^−3^ sub/site/year^56^. The findings from these studies^44^,^54^,^55^,^56^ suggest that the presence of vaccine pressure may drive antigenic drift of AIVs in domestic poultry. In our study, antigenic cartography showed that the average antigenic distance among 7 poultry-origin H7 isolates was 1.13 units (SD, 0.71 unit). The limited antigenic diversity may be attributable to the absence of large-scale vaccination programs against H7 AIVs in North America, with the exception of Mexico. The estimated mean evolution rate for the H7 gene of AIVs from domestic poultry in the United States was 5.34 × 10^−3^ sub/site/year. This rate is significantly lower than that found in the presence of a vaccination program but similar to that found in the absence of vaccine program.

Adaptations are required when IAVs are transmitted across species, including from waterfowl to land-based birds (e.g., chickens and turkeys)^57^. HA protein plays an important role in host-cell recognition, and mutation in HA protein has been identified as a major determinant of host shift to domestic poultry. Previous research identified 2 amino acid substitutions in the HA1 protein for H7 AIVs after their introduction from wild birds to domestic poultry^20^. Furthermore, a 1997 study showed that evolution of the HA gene could increase significantly after introduction into domestic poultry^58^; the evolution rate for the HA gene of H5 AIVs increased significantly after introduction into domestic poultry in Mexico^58^. Our results showed the same trend for the H7 gene of AIVs from North America: the HA gene of poultry-origin H7 AIVs evolves faster than that of waterfowl-origin viruses. Moreover, results showed that the evolution rate for the H7 gene was faster during 1994–1996 than in subsequent years. The rapid evolution during 1994–1996 was mirrored by an 8–amino acid deletion in 1996 at positions 212–219 in the HA1 protein. This deletion removes 5 of 6 consecutive amino acids in part of the receptor binding site. These findings suggest that the H7 gene underwent rapid adaptation in the receptor binding domain after introduction into domestic poultry.

Of interest, position 189 (H3 numbering; H7 numbering, 180) in the HA1 protein was identified as being under positive selection pressure, and this site is located in the receptor binding site for H7 IAVs^59^. Extensive polymorphisms, representing 6 distinct amino acids (A, D, I, N, S, and T), are present at position 189. A previous study showed that propagation of human H1N1 influenza virus in embryonated chicken eggs could cause the substitution E189K at the HA1 protein^60^. R189K was found to have contributed to the antigenic drift of H3N2 IAV^61^, and position 189 plays an important role for the antigenicity of H3 equine influenza virus^62^. Another study showed that single amino acid changes at position A186D (H3 numbering, 189) could increase yield of A/California/7/09(H1N1) virus in eggs^63^. Most influenza viruses recovered from avian samples have been propagated by using chicken embryonated eggs, and a previous study demonstrated frequent adaptation of H1N1 waterfowl-origin AIVs during propagation in embryonated eggs^64^. The receptors in chicken embryonated eggs and those in the mallard gastrointestinal track are not exactly the same. In addition, expression of sialic acids showed substantial host-specific distinctions among avian species. Expression of α2,3-linked sialic acids and α2,6-linked sialic acids were observed in chicken trachea, whereas α2,3-linked sialic acids were predominant in ducks^65^,^66^. Thus, it is possible that the polymorphisms and positive selection detected in position 189 in the HA1 protein were due to viral propagation in embryonated eggs.

Our phylogenetic analysis identified the 2-way intercontinental flow of the AIV H7 gene through the migration of wild birds. At least 2 independent introductions (in 1992 and 1994) from the Eurasian genetic pool to the North American genetic pool were identified. These introductions were not observed in earlier studies with smaller datasets^67^,^68^. Early research detected a subtype H6 IAV in the United States with an HA gene derived from the Eurasian gene pool; the virus subsequently caused an outbreak among poultry in California during 2000–2002^69^. The introduced Eurasian H6 virus has led to the replacement of the endemic H6 AIVs in North America. Recently, outbreaks of novel H5 HPAI viruses have been detected in the United States and Canada. These viruses originated from a wholly Eurasia-origin H5N8 virus introduced to North America by migratory waterfowl through Beringia in 2014^70^. Two novel reassortants (H5N2 and H5N1) were generated by reassortments with viruses circulating in North America. The Beringian Crucible, including Alaska and the Russian Far East, serves as a common breeding area for diverse bird species from Asia and North America. This area provides an ideal environment for reassortment between IAVs carried by migratory birds from distinct gene pools, and it allows for the intercontinental transfer of gene segments or whole virus. Gene flow increases the diversity in individual genetic pools, and diversity could enhance the risk of generating novel strains that can spread more efficiently among birds and even cross the species barrier and cause transmission to mammals.

Our evolutionary analysis revealed frequent reassortment of 6 internal genes of H7 AIVs in the NA-WB lineage; no clear link was identified for any specific gene segment. The internal gene constellation was diverse and transient. This finding was concordant with those from a 2008 study conducted with a smaller dataset of AIVs and without differentiating the HA subtype^71^. However, our finding of a diverse and transient gene constellation differed from the finding of a limited number of stable internal gene cassettes for IAVs adapted to mammals. The 8 gene segments of IAVs could evolve differently due to different selection pressures. The evolution of the internal gene segments may be determined by functional constraints rather than immune pressure for the genes coded for the 2 surface proteins. In wild birds, internal protein genes are highly conserved on the amino acid level, and they could form a large pool of functionally equivalent gene segments. Such a pool would allow frequent reassortment because the exchange of functionally equivalent gene segments is not likely to attenuate the relative fitness of the reassorted viruses. For mammalian adapted IAVs, certain internal gene constellations were suggested to confer a selective advantage to the virus^72^. Mutations may be acquired for internal genes after adaptation to a new host, and those mutations would separate the viruses from those in the natural reservoir. The protein–protein interaction could force the co-evolution of these gene segments and maintain the existence of a specific internal gene constellation.

In summary, our findings demonstrate a limited antigenic diversity among contemporary subtype H7 avian-origin IAVs from North America. H7 IAVs from migratory waterfowl, domestic poultry, and a seal, which together represent a diverse geographic and temporal coverage, were included in this study. The limitation of this study was the small number of H7N3 isolates from the ongoing AIV outbreak in Mexico. Additional studies on a longitudinal collection of H7N3 viruses from the outbreak in Mexico would add to our understanding of the influence vaccination programs have on genetic and antigenic evolutionary dynamics. H7N9 viruses are enzootic in China^17^, but the antigenic properties of these viruses have not been characterized. An antigenic comparison of H7 AIVs from the United State and those from outbreaks in other regions, including China and Europe, is also lacking. Due to the possible transmission of subtype H7 AIVs across regions and continents, constant monitoring of emerging H7 AIVs in North America must continue.

## Additional Information

**How to cite this article**: Xu, Y. *et al.* Limited Antigenic Diversity in Contemporary H7 Avian-Origin Influenza A Viruses from North America. *Sci. Rep.* **6**, 20688; doi: 10.1038/srep20688 (2016).

## Supplementary Material

Supplementary Information

Supplementary Dataset

## Figures and Tables

**Table 1:**
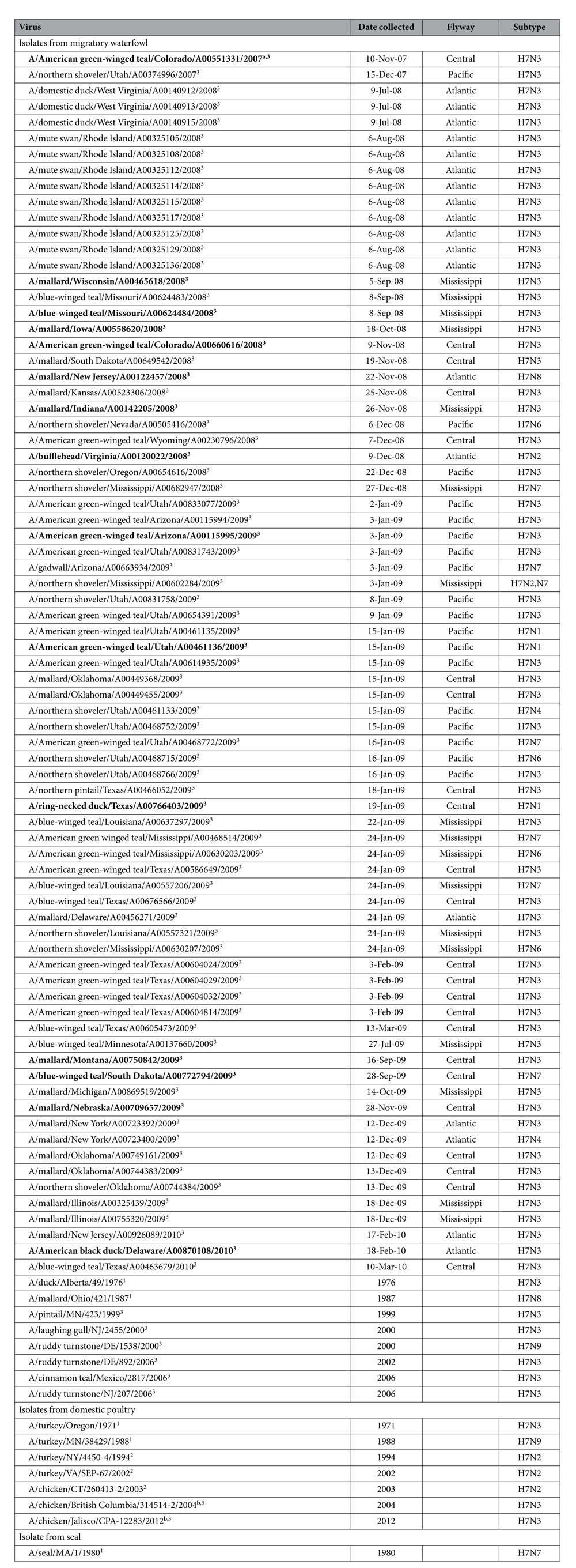
List of subtype H7 AIVs isolated in North America that were subjected to antigenic characterization.

Isolates were grouped according to their genetic clustering in Fig. 1A. ^1^indicates virus in cluster I, ^2^indicates virus in cluster II, and ^3^indicates virus in cluster III. ^a^Viruses used to generated reference antisera are in bold. ^b^Highly pathogenic avian influenza virus.

**Table 2:** Sequence identity of HA1 protein of subtype H7 AIVs from distinct genetic clusters from North America.

% nucleotide/amino acid identity with viruses in genetic cluster			
Genetic cluster	I	II	III
I	94.0/96.8	88.0/90.4	89.4/94.9
II		96.1/95.7	89.5/92.0
III			95.6/98.0

**Table 3:** Estimated rates of nucleotide substitution and time of most recent common ancestors for subtype H7 AIVs from distinct genetic clusters from North America.

Genetic cluster	Model	Substitution rate ( × 10^−3^ substitutions/site/year)			tMRCA (calendar year)		
		Mean	95% HPD^a^	ESS^b^	Mean	95% HPD	ESS
I	SRD06-UCL–Skyline^c^	5.11	3.94–6.25	347	1969	1963–1970	289
II	SRD06-UCL–Skyline	5.34	4.68–6.11	693	1993	1990–1993	406
II^d^	SRD06-UCL–Skyline	4.90	4.19–5.67	856	1996	1995–1996	1070
III	SRD06-UCL–Skyline	4.84	4.34–5.31	389	1992	1991–1992	780
III^e^	SRD06-UCL–Skyline	4.60	4.10–5.11	299	1992	1990– 1992	693

^a^HPD: highest probability density.

^b^Effective sample size.

^c^SRD06: HKY substitution model; UCL: uncorrelated lognormal molecular clock, Skyline Coalescent Bayesian Skyline tree model.

^d^Includes only viruses isolated after 1996.

^e^Includes only viruses isolated from waterfowl.

**Table 4:** Cross-hemagglutination inhibition data obtained for representative subtype H7 AIVs against chicken serum^a^.

	Titer to chicken antiserum generated against 15 selected isolates														
Virus	BUFF120022	MALL122457	MALL465618	AGWT551331	ABDU870108	MALL750842	MALL709657	AGWT115995	MALL558620	MALL142205	AGWT660616	AGWT461136	BWTE624484	RNDU766403	BWTE772794
A/turkey/Oregon/1971	160	320	320	640	640	160	160	320	320	320	40	40	160	320	640
A/duck/Alberta/49/1976	80	320	160	160	160	160	160	80	320	320	160	160	80	160	320
A/seal/MA/1/1980	40	160	80	160	80	40	40	40	80	80	80	80	20	80	160
A/mallard/Ohio/421/1987	40	160	80	80	80	40	40	40	80	160	80	80	20	80	160
A/turkey/MN/38429/1988	40	40	80	80	80	40	40	40	40	80	40	80	40	80	80
A/turkey/NY/4450-4/1994	80	80	80	160	80	80	80	40	80	80	160	80	40	80	40
A/pintail/MN/423/1999	80	160	320	640	320	160	80	80	320	320	160	80	80	160	320
A/laughing gull/NJ/2455/2000	10	10	320	640	320	160	320	160	320	320	320	640	160	320	640
A/turkey/VA/SEP-67/2002	40	40	80	80	80	80	80	80	80	80	40	80	40	80	80
A/chicken/CT/260413-2/2003	40	40	40	80	40	40	20	20	40	40	40	20	20	40	40
A/chicken/British Columbia/314514-2/2004	80	160	160	160	160	160	80	160	160	160	80	80	80	160	320
A/cinnamon teal/Mexico/2817/2006	80	160	320	320	320	160	160	160	320	320	320	320	160	320	320
A/northern shoveler/Utah/A00374996/2007	80	160	160	160	160	160	80	40	80	160	80	320	40	320	320
A/bufflehead/Virginia/A00120022/2008	80	80	80	160	320	80	80	80	80	640	160	320	80	160	320
A/American green-winged teal/Arizona/A00115994/2009	40	80	80	160	80	80	80	40	80	160	40	160	40	160	160
A/blue-winged teal/Texas/A00463679/2010	80	320	160	160	160	80	80	40	160	320	80	320	80	160	320
A/chicken/Jalisco/CPA-12283/2012	40	40	80	80	80	40	40	40	40	80	40	80	40	80	160

^a^The 17 H7 selected AIVs represent the maximal temporal coverage of 93 viruses isolated during 1971–2012. The complete data are shown in appendix Table S1. Abbreviations: BUFF120022, A/bufflehead/VA/A00120022/2008(H7N2); MALL122457, A/mallard/NJ/A00122457/2008(H7N8); MALL465618, A/mallard/WI/A00465618/2008(H7N3); AGWT551331, A/American green winged teal/CO/A00551331/2007(H7N3); ABDU870108, A/black duck/DE/A00870108/2010(H7N3); MALL750842, A/mallard/MT/A00750842/2009(H7N3); MALL709657, A/mallard/NE/A00709657/2009(H7N3); AGWT115995, A/American green winged teal/AZ/A00115995/2009(H7N7); MALL558620, A/mallard/IA/A00558620/2008(H7N3); MALL142205, A/mallard/IN/A00142205/2008(H7N3); AGWT660616, A/American green-winged teal/Colorado/A00660616/2008(H7N3); AGWT461136, A/American green-winged teal/Utah/A00461136/2009(H7N1); BWTE624484, A/blue-winged teal/Missouri/A00624484/2008(H7N3); RNDU766403, A/ring-necked duck/Texas/A00766403/2009(H7N1); BWTE772794, A/blue-winged teal/South Dakota/A00772794/2009(H7N7).

**Table 5:** Selective pressure in HA1 protein of subtype H7 AIVs from North America.

Genetic cluster, model	d_N_/d_S_	Positively selected sites (probability)
I		
M1a	0.1048	
M7	0.0999	
II		
M1a	0.2154	
M7	0.1875	
II^a^		
M1a	0.2121	
M7	0.1821	
III		
M1a	0.1407	
M7	0.1232	
III^b^		
M1a	0.1242	
M8	0.1086	189^c^ (99.7%)

^a^Include only viruses isolated after 1996.

^b^Include only viruses isolated from waterfowl.

^c^H3 numbering.

**Table 6:** Amino acid polymorphism at position 189 (H3 numbering) in HA1 protein for subtype H7 AIVs from North America and Eurasia.

Genetic group	Species	No. of sequences	Relevant amino acids	No. of corresponding amino acids
NA I^a^	L^b^	16	T	16
	W	32	A/N/T	5/4/23
NA II	L	221	I/S	1/220
	W	15	S	15
NA III	L	39	A/N/T	1/6/32
	W	351	A/D/I/N/T	11/48/1/20/271
NA human isolates	H^e^	3	S/T	1/2
EA^c^	L/W	1,118	A/G/N/T	670/1/1/446

^a^Genetic cluster I in North American lineage.

^b^L, land-based poultry; W, waterfowl; H, human.

^c^Eurasian lineage.
