# Supplementary material for: Limited Antigenic Diversity in Contemporary H7 Avian-Origin Influenza A Viruses from North America
Source: Sci Rep. 2016 Feb 9;6:20688. doi: 10.1038/srep20688 (PMC4746648; doi:10.1038/srep20688)

Supplementary Information

Limited Antigenic Diversity in Contemporary H7 Avian-Origin Influenza A Viruses from North America

Running title: Antigenic and genetic characterization of H7 influenza A viruses from North America

Yifei Xu,[a] Elizabeth Bailey,[a] Erica Spackman,[b] Tao Li,[c] Hui Wang,[a] Li-Ping Long,[a] John A. Baroch,[d] Fred L. Cunningham,[e] Xiaoxu Lin,[c] Richard G. Jarman,[c] Thomas J. DeLiberto,[d] and Xiu-Feng Wan[a#]

Department of Basic Sciences, College of Veterinary Medicine, Mississippi State University, Mississippi State, Mississippi, the United States[a]; Exotic and Emerging Avian Viral Diseases Unit, Southeast Poultry Research Laboratory, US Department of Agriculture Agricultural Research Service, Athens, Georgia, the United States[b]; Viral Diseases Branch, Walter Reed Army Institute of Research, Silver Spring, Maryland, the United States[c]; National Wildlife Research Center, Wildlife Services, Animal and Plant Health Inspection Service, US Department of Agriculture, Fort Collins, Colorado, the United States[d]; Mississippi Field Station, National Wildlife Research Center, Wildlife Services, Animal and Plant Health Inspection Service, US Department of Agriculture, Mississippi State, Mississippi, the United States[e].

# Address correspondence to Xiu-Feng Wan, Department of Basic Sciences, College of Veterinary Medicine, Mississippi State University, 240 Wise Center Dr.,
Mississippi State, MS 39762, USA; Phone: (662)325-3559; Fax: (662)325-3884; E-mail: wan@cvm.msstate.edu

Supplementary Figure and Table

Fig S1. Phylogenetic analysis of HA1 nucleotide sequences of H7 avian influenza viruses. The tree was inferred by using the maximum likelihood method implemented in RAxML v8.1.17. Bootstrap values were estimated from 1,000 resamplings of the sequence data, and they are shown adjacent to selected nodes. Clusters and clades are indicated by the black bar on the right. Isolates subjected to antigenic characterization are color-coded according to genetic cluster: red indicates cluster I, green indicates cluster II, and blue indicates cluster III. Purple represents intercontinental gene flow between North American and Eurasian genetic pool. GenBank accession numbers are shown at the end of strain names. Am = North American lineage. Eu = Eurasian lineage.

Fig S2. Amino acid variations in antibody binding sites of H7 AIVs selected for antigenic characterization. The conservation analysis were characterized using WebLogo 3 webserver [1]. The corresponding antibody binding sites in influenza H3 protein are indicated by A, B, C, D, and E at the bottom.

Table S1. Cross-HI data obtained for testing H7 AIVs against representative chicken sera. The homologous titers were underlined.

Virus	Titer to chicken antiserum														
	BUFF120022	MALL122457	MALL465618	AGWT551331	ABDU870108	MALL750842	MALL709657	AGWT115995	MALL558620	MALL142205	AGWT660616	AGWT461136	BWTE624484	RNDU766403	BWTE772794
A/bufflehead/Virginia/A00120022/2008	80	80	80	160	320	80	80	80	80	640	160	320	80	160	320
A/mallard/New Jersey/A00122457/2008	80	80	80	80	160	80	40	20	80	160	80	80	40	160	320
A/mallard/Wisconsin/A00465618/2008	80	160	160	160	160	160	40	40	80	160	80	320	80	320	320
A/American green-winged teal/Colorado/A00551331/2007	80	160	80	80	160	80	40	20	40	160	80	160	40	160	320
A/American black duck/Delaware/A00870108/2010	80	80	80	160	160	80	80	80	160	160	80	160	40	320	640
A/mallard/Montana/A00750842/2009	160	160	320	320	320	320	160	160	160	320	80	640	40	320	640
A/mallard/Nebraska/A00709657/2009	80	320	80	80	160	80	160	160	80	160	160	320	80	320	640
A/American green-winged teal/Arizona/A00115995/2009	20	80	40	40	80	40	40	40	80	80	40	80	20	80	160
A/mallard/Iowa/A00558620/2008	20	80	40	40	40	40	40	20	40	80	40	80	20	80	160
A/mallard/Indiana/A00142205/2008	160	160	160	160	160	160	160	160	40	80	40	160	40	160	320
A/American green winged teal/Colorado/A00660616/2008	40	160	40	80	160	40	40	40	40	160	40	320	40	320	320
A/American green-winged teal/Utah/A00461136/2009	20	160	160	160	160	40	40	40	40	160	40	160	40	80	160

A/blue winged teal/Missouri/A00624484/2008	160	160	160	160	160	160	160	160	80	320	160	640	160	640	1280
A/ring-necked duck/Texas/A00766403/2009	40	160	80	80	160	80	40	40	40	160	80	160	40	160	320
A/blue-winged teal/South Dakota/A00772794/2009	160	640	320	320	320	320	320	320	640	640	320	640	160	640	640
A/seal/MA/1/1980	40	160	80	160	80	40	40	40	80	80	80	80	20	80	160
A/chicken/CT/260413-2/2003	40	40	40	80	40	40	20	20	40	40	40	20	20	40	40
A/chicken/British Columbia/314514-2/2004	80	160	160	160	160	160	80	160	160	160	80	80	80	160	320
A/turkey/Oregon/1971	160	320	320	640	640	160	160	320	320	320	40	40	160	320	640
A/turkey/MN/38429/1988	40	40	80	80	80	40	40	40	40	80	40	80	40	80	80
A/chicken/Jalisco/CPA-12283/2012	40	40	80	80	80	40	40	40	40	80	40	80	40	80	160
A/turkey/VA/SEP-67/2002	40	40	80	80	80	80	80	80	80	80	40	80	40	80	80
A/turkey/NY/4450-4/1994	80	80	80	160	80	80	80	40	80	80	160	80	40	80	40
A/duck/Alberta/49/1976	80	320	160	160	160	160	160	80	320	320	160	160	80	160	320
A/pintail/MN/423/1999	80	160	320	640	320	160	80	80	320	320	160	80	80	160	320
A/laughing gull/NJ/2455/2000	10	10	320	640	320	160	320	160	320	320	320	640	160	320	640
A/ruddy turnstone/DE/1538/2000	40	80	80	80	80	40	40	80	160	160	40	80	20	80	160
A/mallard/Ohio/421/1987	40	160	80	80	80	40	40	40	80	160	80	80	20	80	160
A/cinnamon teal/Mexico/2817/2006	80	160	320	320	320	160	160	160	320	320	320	320	160	320	320
A/ruddy turnstone/DE/892/2006	20	40	40	40	80	20	20	20	40	80	40	40	10	40	80
A/ruddy turnstone/NJ/207/06	20	40	40	40	40	40	20	20	40	80	40	40	20	40	80
A/American green-winged teal/Arizona/A00115994/2009	40	80	80	160	80	80	80	40	80	160	40	160	40	160	160

A/blue-winged teal/Minnesota/A00137660/2009	80	160	80	80	80	80	80	40	320	320	80	160	40	320	320
A/domestic duck/West Virginia/A00140912/2008	40	80	80	80	80	40	160	20	40	80	40	160	40	160	320
A/domestic duck/West Virginia/A00140913/2008	80	80	80	80	80	80	40	40	40	80	80	80	40	80	80
A/domestic duck/West Virginia/A00140915/2008	40	80	80	80	80	80	40	20	40	160	80	160	40	160	320
A/American green-winged teal/Wyoming/A00230796/2008	40	80	80	80	80	80	40	40	80	160	80	160	40	160	320
A/mute swan/Rhode Island/A00325105/2008	160	160	160	80	160	40	40	40	40	160	40	160	20	160	320
A/mute swan/Rhode Island/A00325108/2008	40	80	40	40	40	20	20	20	20	40	40	80	20	40	40
A/mute swan/Rhode Island/A00325112/2008	20	40	40	40	40	20	20	20	80	80	40	80	20	40	160
A/mute swan/Rhode Island/A00325114/2008	20	40	40	40	40	20	40	40	80	80	40	40	20	80	160
A/mute swan/Rhode Island/A00325115/2008	160	160	40	80	160	40	40	40	40	160	40	80	20	80	160
A/mute swan/Rhode Island/A00325117/2008	40	80	40	40	40	20	20	20	20	40	20	80	20	40	80
A/mute swan/Rhode Island/A00325125/2008	40	80	40	40	80	40	40	20	40	160	40	160	20	160	160
A/mute swan/Rhode Island/A00325129/2008	20	40	40	40	40	20	20	20	40	40	20	80	20	80	40
A/mute swan/Rhode Island/A00325136/2008	40	80	80	80	80	40	40	20	40	80	40	80	20	80	80

A/mallard/Illinois/A00325439/2009	40	80	80	80	80	40	40	40	40	80	40	80	40	80	80
A/northern shoveler/Utah/A00374996/2007	80	160	160	160	160	160	80	40	80	160	80	320	40	320	320
A/mallard/Oklahoma/A00449368/2009	80	160	80	80	80	40	40	40	80	160	40	160	20	160	160
A/mallard/Oklahoma/A00449455/2009	16	20	20	20	20	20	20	20	20	20	20	20	20	40	80
A/mallard/Delaware/A00456271/2009	80	320	320	320	320	160	80	80	160	320	80	320	80	160	320
A/northern shoveler/Utah/A00461133/2009	80	80	80	80	80	80	80	80	80	80	80	80	80	80	80
A/American green-winged teal/Utah/A00461135/2009	80	80	80	80	80	80	80	80	80	80	80	80	80	80	80
A/blue-winged teal/Texas/A00463679/2010	80	320	160	160	160	80	80	40	160	320	80	320	80	160	320
A/northern pintail/Texas/A00466052/2009	40	80	40	80	160	40	40	20	40	80	40	80	20	160	160
A/American green-winged teal/Mississippi/A00468514/2009	160	160	320	320	320	160	320	160	160	320	160	320	80	320	640
A/northern shoveler/Utah/A00468715/2009	20	20	20	20	40	20	20	20	40	40	20	20	20	20	40
A/northern shoveler/Utah/A00468752/2009	80	80	80	80	80	80	80	80	80	160	64	160	40	160	160
A/northern shoveler/Utah/A00468766/2009	80	160	80	80	160	80	40	40	20	160	40	320	40	160	320
A/American green-winged teal/Utah/A00468772/2009	40	80	80	80	80	40	40	40	160	160	80	160	40	320	320

A/northern shoveler/Nevada/A00505416/2008	40	80	80	80	80	40	40	80	40	80	40	80	40	80	80
A/mallard/Kansas/A00523306/2008	80	160	80	160	160	80	40	40	40	160	80	160	40	320	320
A/blue-winged teal/Louisiana/A00557206/2009	160	160	160	160	160	160	40	80	160	160	40	80	40	160	160
A/northern shoveler/Louisiana/A00557321/2009	40	80	40	40	40	40	40	20	320	320	160	320	40	160	320
A/American green-winged teal/Texas/A00586649/2009	40	80	80	80	80	40	40	40	20	80	40	80	20	160	160
A/northern shoveler/Mississippi/A00602284/2009	160	640	320	640	640	160	320	160	160	320	320	320	80	320	320
A/American green-winged teal/Texas/A00604024/2009	80	160	160	160	160	80	80	40	40	160	80	160	40	160	320
A/American green-winged teal/Texas/A00604029/2009	40	80	80	80	80	40	40	40	40	80	40	80	40	80	80
A/American green-winged teal/Texas/A00604032/2009	320	160	320	320	320	160	160	80	320	320	80	640	80	640	640
A/American green-winged teal/Texas/A00604814/2009	160	320	160	320	160	160	80	80	320	320	80	320	80	160	160
A/blue-winged teal/Texas/A00605473/2009	20	80	40	40	40	20	20	20	20	40	20	80	20	80	80
A/American green-winged teal/Utah/A00614935/2009	160	320	160	160	320	160	160	160	320	320	80	640	80	320	320
A/blue-winged teal/Missouri/A00624483/2008	160	80	40	40	160	40	20	20	20	160	20	160	40	160	320

A/American green-winged teal/Mississippi/A00630203/2009	20	160	160	160	160	160	40	20	160	320	80	160	40	160	320
A/blue-winged teal/Louisiana/A00637297/2009	40	80	40	40	40	20	20	20	40	80	160	160	20	160	320
A/mallard/South Dakota/A00649542/2008	80	160	80	80	160	80	40	20	40	160	80	160	40	160	320
A/American green-winged teal/Utah/A00654391/2009	80	80	80	80	80	80	40	40	40	80	80	80	40	80	80
A/northern shoveler/Oregon/A00654616/2008	80	320	80	80	160	80	80	160	160	160	80	320	80	640	2048
A/gadwall/Arizona/A00663934/2009	80	80	80	160	160	80	40	20	40	160	80	80	40	80	160
A/blue-winged teal/Texas/A00676566/2009	40	320	80	80	80	80	80	80	80	160	160	320	40	320	320
A/northern shoveler/Mississippi/A00682947/2008	160	80	320	320	160	320	160	160	640	320	160	320	80	640	640
A/mallard/New York/A00723392/2009	20	80	40	40	40	20	20	20	40	80	20	80	20	80	160
A/mallard/New York/A00723400/2009	40	80	80	160	160	80	40	40	64	160	80	80	40	160	160
A/mallard/Oklahoma/A00744383/2009	80	160	80	160	160	80	160	160	320	160	80	320	80	320	320
A/northern shoveler/Oklahoma/A00744384/2009	80	320	80	80	160	80	80	80	320	160	80	160	40	160	320
A/mallard/Oklahoma/A00749161/2009	40	80	40	40	80	40	40	40	40	80	40	80	20	80	80

A/mallard/Illinois/A00755320/2009	80	320	160	160	320	160	80	80	160	320	80	80	80	640	640
A/American green-winged teal/Utah/A00831743/2009	160	160	160	160	160	160	40	40	40	320	80	320	80	320	640
A/northern shoveler/Utah/A00831758/2009	80	160	320	320	160	160	160	160	160	640	80	640	80	320	640
A/American green winged teal/Utah/A00833077/2009	40	160	160	40	160	160	40	40	20	160	20	160	40	1280	1280
A/mallard/Michigan/A00869519/2009	80	320	80	80	80	80	80	80	320	160	80	320	40	160	160
A/mallard/New Jersey/A00926089/2010	80	160	40	80	80	40	40	20	40	80	64	160	20	80	80
A/northern shoveler/Mississippi/A00630207/2009	160	320	320	320	320	160	80	80	80	320	160	320	80	320	640

Abbreviations: BUFF120022, A/bufflehead/VA/A00120022/2008(H7N2); MALL122457, A/mallard/NJ/A00122457/2008(H7N8); MALL465618, A/mallard/WI/A00465618/2008(H7N3); AGWT551331, A/American green winged teal/CO/A00551331/2007(H7N3); ABDU870108, A/black duck/DE/A00870108/2010(H7N3); MALL750842, A/mallard/MT/A00750842/2009(H7N3); MALL709657, A/mallard/NE/A00709657/2009(H7N3); AGWT115995, A/American green winged teal/AZ/A00115995/2009(H7N7); MALL558620, A/mallard/IA/A00558620/2008(H7N3); MALL142205, A/mallard/IN/A00142205/2008(H7N3); AGWT660616, A/American green-winged teal/Colorado/A00660616/2008(H7N3); AGWT461136, A/American green-winged teal/Utah/A00461136/2009(H7N1); BWTE624484, A/blue-winged teal/Missouri/A00624484/2008(H7N3); RNDU766403, A/ring-necked duck/Texas/A00766403/2009(H7N1); BWTE772794, A/blue-winged teal/South Dakota/A00772794/2009(H7N7). Isolates' name was colored-coded according to their genetic clustering in figure 1A. Red indicates virus in cluster I, green indicates virus in cluster II, and blue indicates virus in cluster III.

REFERENCES

1 Crooks, G. E., Hon, G., Chandonia, J.-M. & Brenner, S. E. WebLogo: a sequence logo generator. *Genome research* **14**, 1188-1190 (2004).

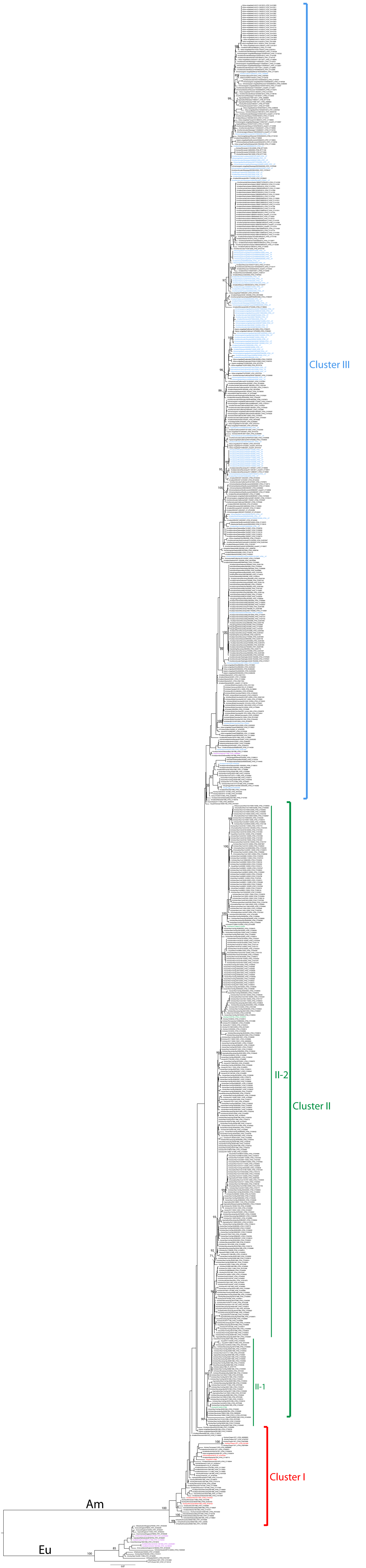

Cluster III
II-2
Cluster II
II-1
Cluster I
Am
Eu

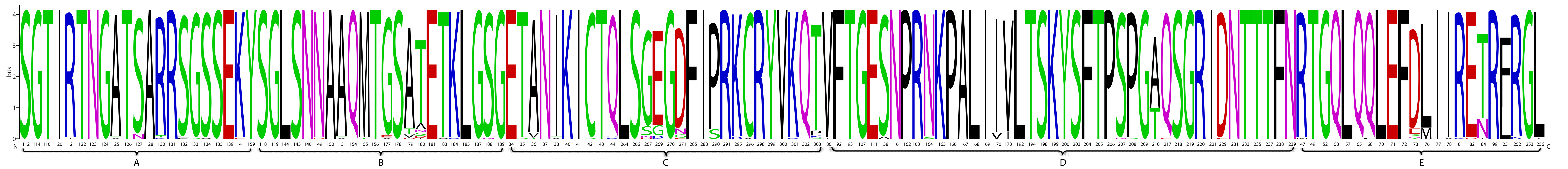

Supplement: Supplementary Information [file srep20688-s1.pdf]
